# Specific Impact of the Layered Nanomodifiers—Graphene Nanoplates, and Na^+^ Montmorillonite on Thermal Degradation of Polylactic Acid: Mechanism and Kinetics

**DOI:** 10.3390/polym18030347

**Published:** 2026-01-28

**Authors:** Sergey Lomakin, Elena Koverzanova, Sergey Usachev, Natalia Shilkina, Anatoliy Khvatov, Natalia Erina, Svetlana Rogovina, Olga Kuznetsova, Valentina Siracusa, Alexander Berlin, Alexey Iordanskii

**Affiliations:** 1 N. N. Semenov Federal Research Center for Chemical Physics Russian Academy of Science, 119991 Moscow, Russia; koverlena@list.ru (E.K.); usachevsv@inbox.ru (S.U.); tashi05@list.ru (N.S.); natalia.erina@mail.ru (N.E.); s.rogovina@mail.ru (S.R.); 123zzz321@inbox.ru (O.K.); berlin@chph.ras.ru (A.B.); 2 Emanuel Institute of Biochemical Physics, Russian Academy of Sciences, 119334 Moscow, Russia; hvatovanatoliy@gmail.com; 3 Department of Chemical Science (DSC), University of Catania, Viale A. Doria 6, 95125 Catania, Italy; vsiracus@dmfci.unict.it

**Keywords:** polylactide, thermal degradation, kinetics modeling, graphene nanoplates, Na-montmorillonite, atomic force microscopy, differential scanning calorimetry, thermogravimetric analysis, gas chromatography–mass spectrometry

## Abstract

The aim of this study is to investigate the impact of layered nanomodifiers with distinct chemical structure and morphology, namely graphene nanoplates (GnP) and sodium montmorillonite (Na-MMT), on thermal degradation of polylactic acid (PLA). The exploration was performed with thermogravimetric analysis (TGA), differential scanning calorimetry (DSC), and pyrolytic gas chromatography–mass spectrometry (PyGCMS). The findings revealed a catalytic effect of Na-MMT on PLA thermal destabilization, manifested in accelerated degradation and the notable change in the composition of pyrolysis products. In contrast, the incorporation of graphene nanoplates into the PLA matrix induced a “barrier effect”: it imposed diffusion limitations on the emission of volatile degradation products during pyrolysis, which enhanced the thermal stability of the PLA/GnP composite and led to quantitative alterations in the distribution of major pyrolysis products. To elucidate the underlying degradation pathways, authors proposed a model kinetic analysis of thermal degradation for both PLA/GnP and PLA/Na-MMT composites. The analysis clearly distinguished the mechanistic differences between the two systems: while Na-MMT promotes catalytic decomposition, GnP primarily acts as the physical barrier retarding mass transport and delaying the thermal degradation development. Good alignment of theoretical model–kinetic predictions with Pyrolysis–GC/MS observations confirms the robustness of the suggested kinetic modeling method.

## 1. Introduction

The polylactides (PLAs), classified as aliphatic polyesters synthesized from bio-based lactic acid, have gained recognition as the viable bio-based polymers proposed as the alternative to conventional petroleum-derived plastics. Although PLA exhibits acceptable mechanical properties and relevant barrier characteristics, their thermal stability falls within the optimal requirements, constituting a major limitation in polymer applications [1,2,3,4,5,6]. To overcome this inherent limitation and expand the area of implementation, PLA is frequently combined with different functional fillers [7,8]. Among these, nanosized carbon materials, such as carbon nanotubes, carbon fibers, graphene, graphene nanoplates (GnP), and layered clays as organically modified MMT, have attracted considerable interest due to their high efficiency [9,10,11,12,13,14,15]. The integration of such fillers into PLA matrices results in the formation of advanced nanocomposite materials. These composites demonstrate superior electrical conductivity, enhanced barrier performance, improved physico-mechanical properties, elevated thermal stability, and lowered flammability [16,17,18,19,20,21]. The exploration of novel applications for such composites constitutes a widespread area of both fundamental and applied studies.

In general, the thermal degradation of PLA involves a complex of chemical processes, including radical-chain depolymerization (unzipping), intermolecular transesterification, yielding monomeric and oligomeric esters, and intramolecular transesterification, leading to oligomer formation [22,23,24,25,26]. These processes were studied in detail in the classical works by McNeill et al. and Kopinke [25,26]. The ester interchange mechanism of PLA degradation leading to the formation of lactides with two lactic acid residues (*n* = 2) was suggested on the grounds of pyrolysis product analysis. On the other hand, the formation of cyclic oligomers with *n* > 2 was explained by the intramolecular transesterification mechanism [26]. In addition to lactides and their cyclic oligomers, such low molecular compounds as CO, CO_2_, acetaldehyde, acrylic acid, and acetone were identified in the pyrolysis products [21,22]. Key studies by Kopinke and McNeill et al. have provided detailed insights into the mechanisms driving these processes [25,26]. The breakdown of polylactic acid (PLA) through the backbiting ester interchange pathway was hypothesized following an analysis of pyrolysis by-products. This pathway leads to the creation of lactides that contain two lactic acid residues (*n* = 2). In addition to lactides and cyclic PLA oligomers, pyrolysis yields a range of other compounds. Among these are carbon monoxide (CO), carbon dioxide (CO_2_), acetaldehyde, acrylic acid, and acetone [26]. To date, scientists have proposed various routes for PLA thermal degradation. These pathways cover both non-radical and radical reaction mechanisms. The notable variants of PLA degradation include random chain scission, depolymerization, as well as intramolecular and intermolecular transesterification reactions [26,27].

Recent research by Usachev et al. [28] revealed dioxolanones as the novel degradation products of thermally degraded PLA. These findings lead to a revised mechanism of PLA thermal degradation that challenges the classical model proposed by Kopinke and McNeil [25,26]. While the traditional mechanism emphasizes non-radical reverse exchange between ester bonds and terminal hydroxyl groups, the new pathway suggests that dioxolanones form when terminal hydroxyl groups react with the α-carbon (adjacent to the carbonyl group), rather than with the ester bond. The research also investigated the effect of reduced graphene oxide (rGO) on the pyrolysis process of polylactide (PLA) composites. Compared to pure PLA, the PLA/rGO pyrolysis products showed a significant reduction in five-membered cyclic dioxolanes, alongside increased levels of lactides and cyclic oligomeric lactides. The authors attribute this shift to rGO restricting the segmental mobility of PLA chains, thereby lowering the likelihood of dioxolane formation via the backbiting mechanism (which involves a six-membered ring transition state). Additionally, the elevated lactide concentration in PLA/rGO may stem from enhanced intramolecular reactions within the PLA chains.

Alongside the analysis of thermal degradation product composition (via pyrolysis chromatography–mass spectrometry), thermokinetic methods relying on TGA data represent an essential component in investigating the PLA thermal degradation mechanism [29,30,31].

Thermal degradation kinetics provide valuable insights into the underlying mechanisms and help the prediction of material behavior under various conditions. Thermogravimetric Analysis (TGA) has emerged as a powerful tool for evaluating thermal degradation kinetics in polymeric materials. This technique enables the determination of key kinetic parameters, including apparent activation energy, pre-exponential factors, and reaction orders. Various models can be employed to analyze thermal degradation kinetics, with isoconversion model-free methods gaining prominence due to their versatility. There are various models that can be used to calculate the activation energy, but the isoconversion model-free methods, such as the Flynn–Wall–Ozawa (FWO), Kissinger–Akahira–Sunose, ASTM, Friedman, and Coats–Redfern methods, are gaining prominence due to their versatility [32,33,34,35,36]. Among these, the Flynn–Wall–Ozawa (FWO) method has been particularly effective in analyzing thermal degradation processes, providing reliable results across multiple studies [34,35].

This approach enables the determination of isoconversional activation energy values without relying on any specific reaction model. Due to this feature, isoconversional techniques are commonly referred to as “model-free” methods. Since thermal degradation processes often involve multiple stages and exhibit complex behavior, a nonlinear regression-based kinetic modeling approach is employed to achieve an accurate description. The modeling workflow begins with an initial kinetic assessment using model-free isoconversion methods. This step aims to define the initial conditions along with the kinetic triplets (i.e., activation energy, pre-exponential factor, and reaction order). The data derived from this preliminary analysis can then serve as input parameters for the subsequent nonlinear approximation procedure. In this work, kinetic studies were carried out using a nonlinear regression-based kinetic modeling approach [37,38].

The study’s central innovation lay in the development and verification of a kinetic modeling methodology. This approach enabled the distinction and quantitative assessment of two distinct mechanisms—catalytic degradation and the physical barrier effect. Crucially, the model demonstrated robust alignment with product analysis data obtained via Py-GC/MS. This combination allowed for a precise quantitative description of the accumulation dynamics of primary PLA thermal degradation products under pyrolysis conditions.

A comprehensive investigation was conducted to elucidate the effects of Na-MMT and graphene nanoplates (GnP) fillers on the thermal degradation characteristics of polylactic acid (PLA) composites. The research program encompassed the following: (1) structural analysis via atomic force microscopy (ASM) and differential scanning calorimetry (DSC) to evaluate filler dispersion and interactions; (2) thermogravimetric analysis (TGA) for thermal stability quantification; (3) pyrolysis gas chromatography–mass spectrometry (Py-GC/MS) for detailed product analysis; and (4) kinetic modeling based on a dual-competing reaction framework. Improving the thermal stability of PLA materials is a key objective, making this study particularly valuable. The development of advanced materials and the expansion of PLA composites’ utility in diverse industries rely heavily on a profound comprehension of PLA degradation, encompassing both its mechanisms and kinetic aspects.

## 2. Materials and Methods

### 2.1. Materials

Polylactide PLA 4043D from Nature Works (Minnetonka, MN, USA) in the form of pellets with diameters of 3 mm (M_w_ = 2.2 × 10^5^, M_n_ = 1.65 × 10^5^, T_m_ = 155 °C, polydispersity index D = M_w_/M_n_ = 1.35, transparency 2.1%) was used as a matrix polymer.

The natural montmorillonite known as Cloisite Na^+^ (CEC: 90–95 meq/100 g) was originally provided by Southern Clay Products (Gonzales, TX, USA). Following the company’s acquisition on 1 October 2013, it is now sold by BYK-Chemie GmbH, Wesel, Germany as BYK Cloisite^®^ Na^+^. Graphene nanoplates (GnP) (XG Sciences, Michigan State University, East Lansing, MI, USA) with diameter *d* = 10 nm, length *l* = 5 μ, relation *l*/*d* = 500, surface area between 120 and 150 m^2^/g, and density *ρ* = 1.8 g/cm^3^ were taken as objects of investigation. Dichloromethane from SHARLAU, Barcelona, Spain (HPLC grade) was used as received.

### 2.2. Preparation of Compositions

Compositions of PLA/Na-MMT and PLA/GnP with 1, 5, and 10 wt.% of additives were obtained by the liquid-phase method. For obtaining mixed compositions with 1, 5, and 10 wt.% of filler (Na-MMT and GnP), PLA4043D (495, 475, and 450 mg, respectively) was dissolved in 25 mL CH_2_Cl_2_ at 22 ± 1 °C for 48 h. To the resulting solution, filler (5, 25, and 50 mg, respectively) was added. The mixture was treated on a sonication bath (35 kHz, 50 W, 30 min, 12 ± 2 °C). The resulting mixture was poured into a *Petri* dish. The solvent was removed at room temperature for 24 h and then at 110 °C for 6 h.

### 2.3. Thermogravimetric Analysis (TGA)

Thermogravimetric analysis (TGA) of the samples was performed on NETZSCH TG 209 F1 Iris thermal balance (NETZSCH-Gerätebau GmbH, Bavaria, Germany) in the temperature range of 25–800 °C at the heating rate of 20°/min in inert atmosphere of Ar with a flow rate of 20 mL/min. Weight of samples were 5 ± 1 mg. The primary criteria for assessing the thermal stability of the compositions under investigation are the onset temperature of thermal degradation (T_on_) according to ASTM E2550 standard test method and the temperature at which the maximum rate of thermal degradation (T_max_) occurs, corresponding to the peak temperature observed in the differential thermogravimetry (DTG) analysis, which is the first derivative of the TGA curve.

### 2.4. Differential Scanning Calorimetry (DSC)

The thermophysical characteristics of PLA, PLA/Na-MMT, and PLA/GnP compositions were studied using the DSC method on a DSC-204 F1 Phoenix calorimeter (NETZSCH-Gerätebau GmbH, Bavaria, Germany) at the heating rate of 10 K/min in an inert atmosphere of Ar in the temperature range of 25–180 °C. The numerical calculations of the enthalpy changes (Δ*H*) derived from integral heat flux curves (DSC curves) of PLA and its compositions were conducted utilizing the NETZSCH Proteus version 4.8.4 software. When studying the morphology of polymers by the DSC method, it is customary to use the repeated heating–cooling mode to remove the “prehistory” of their formation and “thermodynamically balance” their original structure. The degree of crystallinity of PLA samples, χ%, was calculated by equation

$$\chi =\frac{\Delta H_{m}-\Delta H_{cc}}{\Delta H_{m}^{100}(1-\alpha )}\times 100\%$$

where ∆*H_m_*—enthalpy of melting; ∆*H_cc_*—enthalpy of “cold” crystallization; α—mass fraction of GnP and Na-MMT fillers (10% by mass.); ∆*H*^100^*_m_*—the theoretical value of the 100% crystalline poly(*L*-lactide) melting enthalpy (93.6 J/g) [39].

### 2.5. Atomic Force Microscopy (AFM)

Structural studies to investigate dispersion and distribution of Na-MMT and GnP fillers in PLA matrix were performed by atomic force microscopy (AFM) in intermittent contact mode (Tapping Mode^TM^) at room temperature using a MultiMode^TM^ scanning probe microscope and a Nanoscope IIIA^TM^ controller (Bruker NanoSurface Inc., Goleta, CA, USA). Before imaging to have an access to the inner structure, the samples were microtomed with a diamond knife at T = −50 °C using the MicroStar 01 device (MSI Computer Corp, City of Industry, CA, USA) to obtain an extremely flat sample surface with low roughness. AFM scanning was performed with silicon probes with a hardness of ~40 Nm and a resonance frequency of ~150 kHz. To enhance the phase contrast, a mode was selected in which the scanning amplitude was 0.4–0.5 of the amplitude of the free oscillation of the probe.

### 2.6. Pyrolysis of PLA, PLA/Na-MMT, and PLA/GnP Compositions

The samples of compositions (1.8 ± 0.1 mg) were subjected to pyrolysis in an inert atmosphere of Ar in a quartz horizontal tubular reactor at the temperature of 400 °C which was controlled with a platinum thermosensitive resistor Pt100 with the accuracy of temperature in the heating zone of sample equal to ±2 °C. The pyrolysis products were removed from the reaction zone with argon flow (15 mL/min) and were collected in methylene chloride (0.6 mL) at 4 °C (bath with cold water). The pyrolysis products were removed from the reaction zone with argon flow (15 mL/min) and were collected in methylene chloride (0.6 mL) at 4 °C (bath with cold water). The analysis of the obtained products was carried out by GC/MS method without further treatment.

### 2.7. Gas Chromatography–Mass Spectrometry (GC/MS)

GC/MS measurements were performed on a gas chromatograph Trace-1310 and ISQ™ Single Quadrupole Mass Spectrometer (Thermo Fisher Scientific Inc., Waltham, MA, USA) as described earlier [38]. MS-spectra of degradation components were identified and interpreted by matching the results with NIST 2011 Mass Spectral library. All detected degradation products were standardized by relative peak area of the particular product (*RPA_i_*) according to the following equation:

$$RPAi=\frac{PAi}{\Sigma PAi}\times 100\%$$

where *PA_i_*—peak area of particular product; Σ*PA_i_*—sum of peak areas of all detected products. Relative peak area of particular product (*RPA_i_*) presents a sum of *RPA_i_* for a corresponding fraction.

### 2.8. Model Thermokinetics

Model kinetic analysis of PLA, PLA/Na-MMT, and PLA/GnP compositions’ thermal degradation was carried out using the NETZSCH Thermokinetics 3.0 software by NETZSCH-Gerätebau GmbH, Bavaria, Germany.

## 3. Results

### 3.1. AFM Characterization

**Figure 1:**
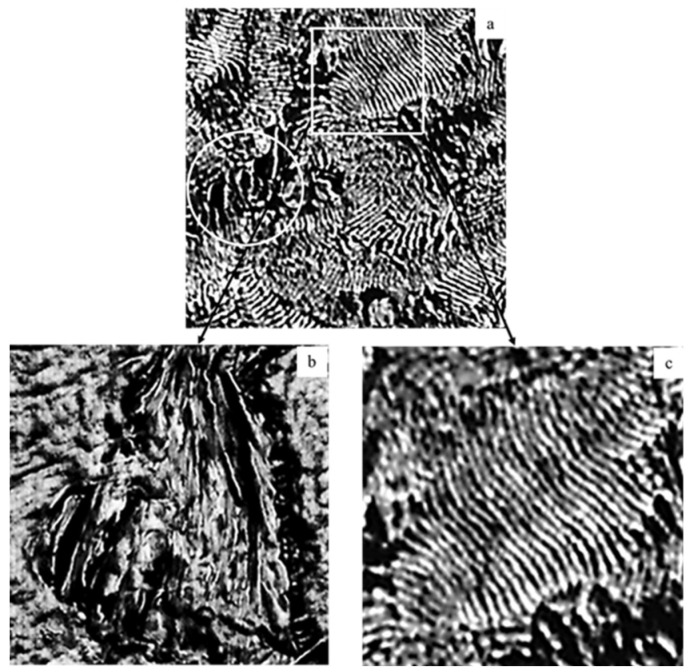
Phase AFM images of the PLA/Na-MMT 5% wt. composite at different magnifications: (**a**) 3 × 3 µm; (**b**,**c**) 1 × 1 µm.

To elucidate in what form Na-MMT and GnP nanofillers were incorporated into the PLA matrix, the results of the high-resolution AFM technique providing the «phase» images are presented in Figure 1. AFM imaging was carried out for the PLA/Na-MMT and PLA/GnP samples containing the same concentrations of the fillers (5 wt.%). It should be noted that the changes in contrast for the AFM images are due to the change in the oscillation phase of the probe when it interacts with regions of the polymer matrix having different mechanical properties due to stiffness or adhesion [40]. In our case, the brightest areas correspond to rigid fillers plates, while the dark ones belong to the PLA matrix.

Images on Figure 1a–c indicate that Na-MMT layered clay are quite evenly distributed in the PLA matrix. The areas with two consistent processes are observed: the first one (Figure 1b) corresponds to the initial intercalation of the matrix material between the layers of Na-MMT clay which are 5–10 nm in thickness and the second one (Figure 1c) shows the areas of filler exfoliation.

**Figure 2:**
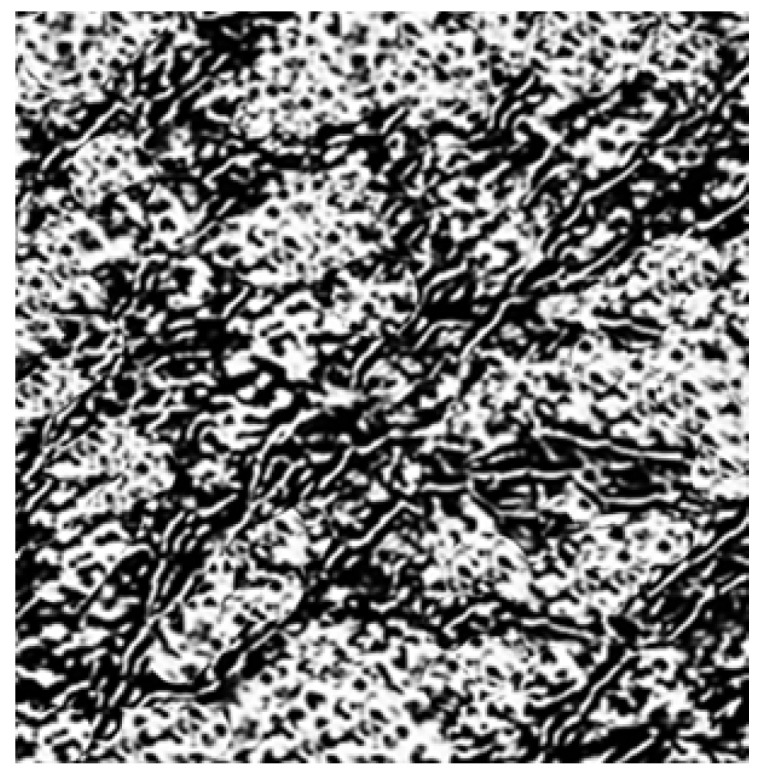
Image (1 × 1 µm) of PLA/GnP composite.

Figure 2 shows the cross-section of the morphological structure in the PLA/GnP composite with interconnected layers of GnP particles with ~10 nm in the thickness. They are preferentially oriented along the flow alignment of polymer matrix during mixing and subsequent pressing at high temperature.

### 3.2. DSC Analysis of PLA/CR PLA/Na-MMT and PLA/GnP Compositions

Differential scanning calorimetry (DSC) was employed to investigate how fillers affect the thermophysical properties and morphological characteristics of the synthesized composites.

**Figure 3:**
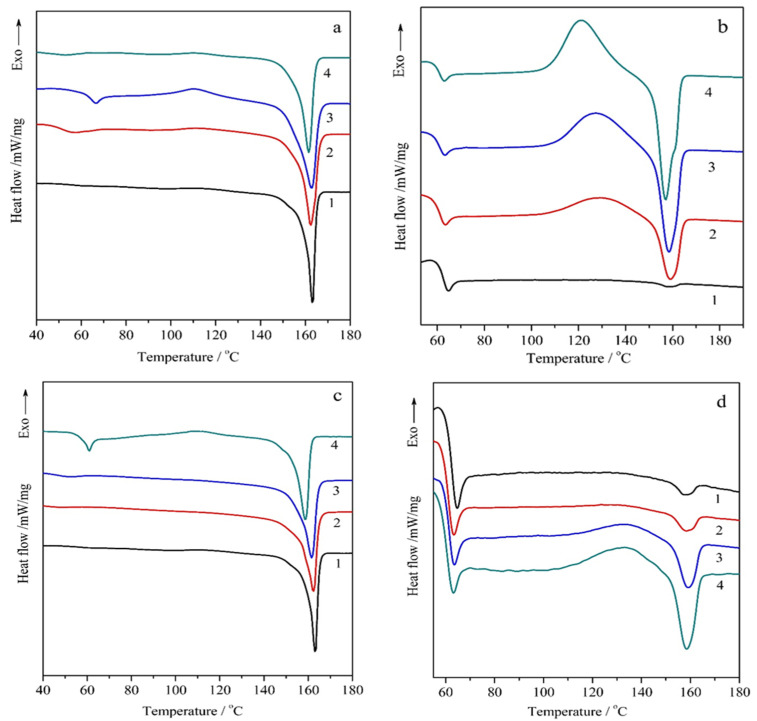
The DSC thermograms depicting both primary and secondary heating for two composite systems: PLA/GnP (**a**,**b**) and PLA/Na-MMT (**c**,**d**). The curves correspond to the following samples: 1—neat PLA; 2—PLA containing 1 wt.% of either Na-MMT or GnP; 3—PLA with 5 wt.% of Na-MMT or GnP; 4—PLA with 10 wt.% of Na-MMT or GnP.

Figure 3 shows the DSC thermograms obtained in a repeated heating–cooling mode for PLA and its various composites containing 1, 5, and 10 wt.% of Na-MMT (Figure 3a,b) and PLA/GnP (Figure 3c,d). The key DSC parameters analyzed include the glass transition temperature (*T*_g_), the peak temperatures for cold crystallization (*T*_cc_), and the melting (*T*_m_). Additionally, the characteristic enthalpies related to cold crystallization (Δ*H_cc_*) and melting (Δ*H_m_*), as well as the degree of crystallization (χ,%), are summarized in Table 1. The analysis of data presented in Figure 3 and Table 1 indicates that a transition from a glassy to an elastic state occurs in all PLA/GnP specimens within the temperature range of 53.9 to 64.6 °C for the first heating and in a narrower temperature range of 61.3 to 62.0 °C for the second heating, respectively. For PLA/Na-MMT compositions, the glass transition occurs within the temperature range of 51.0 to 66.4 °C for the first heating and in a narrower temperature range of 60.8 to 61.2 °C for the second heating, respectively.

At temperatures exceeding 110.5 °C, all PLA compositions exhibited an exothermic effect associated with “cold crystallization” (Table 1). It is important to highlight that a distinct double endothermic melting peak is observed during second heating in the profile of PLA/GnP 10 wt.% composition, as illustrated in Figure 3b and Table 2. These peaks are characteristic of *α*-ordered (orthorhombic) and *α*′-limit disordered (hexagonal) crystalline forms of PLA [41]. The analysis of the primary heating via the DSC technique presented in Table 1 shows that the PLA/Na-MMT 10 wt.% and PLA/GnP 10 wt.% samples exhibit the highest degree of crystallinity for all the samples, measuring 39.7% and 34.2%, respectively.

The results obtained indicate that Na-MMT particles, and to a lesser extent GnP ones, can act as the effective nucleating agents promoting PLA crystallization in the composites. Comparative DSC analysis of the second heating cycle for PLA, PLA/Na-MMT, and PLA/GnP composites revealed that an increase in the filler concentration leads to the marked growth in Δ*H_cc_* relative to the first heating, indicating a nucleating effect of the fillers. Furthermore, the degree of crystallinity dropped more than tenfold upon reheating (Table 1).

### 3.3. Thermogravimetric Analysis of PLA/Na-MMT and PLA/GnP Compositions

In this study, the effect of Na-MMT and GnP additives on the thermal stability of PLA was investigated. As already noted in the introduction, GTP and Na-MMT fillers have different effects on the thermal degradation of PLA. GnP acts as a stabilizing agent in PLA composites, increasing thermal stability as evidenced by higher onset degradation temperatures (T_on_) and maximum degradation temperatures (T_max_). This improvement is attributed to the strong interactions between GnP and PLA, which hinder polymer chain breakdown during heating. Additionally, GnP can act as a barrier to heat penetration, reducing mass loss.

**Figure 4:**
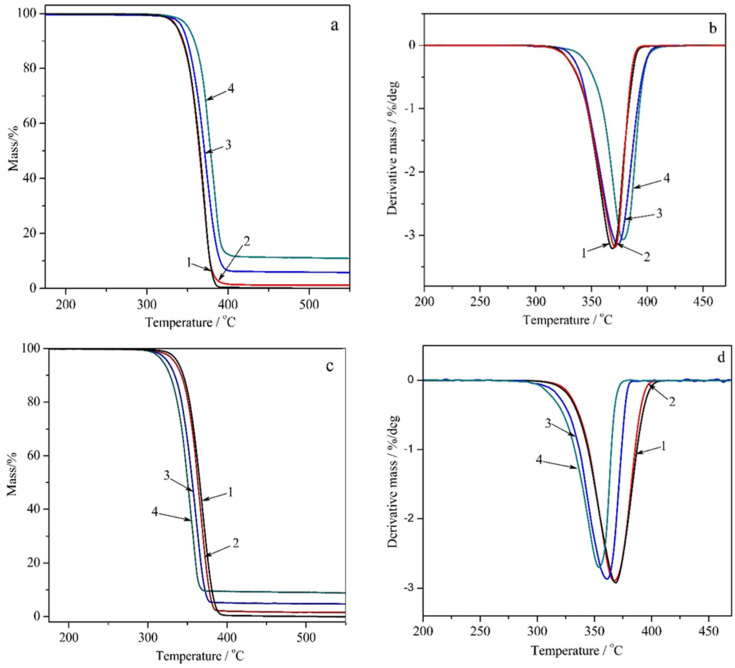
Thermogravimetric analysis representing TG (**a**) and DTG curves (**b**) for PLA/GnP compositions, as well as TG (**c**) and DTG curves (**d**) for PLA/Na-MMT ones where 1—neat PLA; 2—PLA containing 1 wt.% of Na-MMT or GnP; 3—PLA with 5 wt.% of Na-MMT or GnP; 4—PLA with 10 wt.% of Na-MMT or GnP.

In contrast, Na-MMT clay often leads to a decrease in PLA’s thermal stability. The filler addition can lower the onset degradation temperature and reduce the temperature at which maximum weight loss occurs. This destabilizing effect may be due to the presence of sodium cations or traces of water molecules located in the clay structure, which can catalyze degradation reactions. The thermogravimetric (TG) curves and their corresponding derivative (DTG) curves obtained in this work are depicted in Figure 4. A summary of the key parameters derived from the TG curves, which are critical for assessing the dynamics of material degradation, is provided in Table 2, where T_on_ (onset) denotes the temperature at which the degradation of the sample commences and T_max_ signifies the temperature at which the degradation rate of the sample reaches its maximum.

The thermal analysis data (Figure 4a–d and Table 2) reveal divergent trends: PLA/GnP composites demonstrate enhanced thermal stability compared to unmodified PLA, with stability increasing monotonically with GnP content (Figure 4a,b, Table 2). In contrast, PLA/Na-MMT composites exhibit reduced thermal stability relative to pure PLA, with stability decreasing as Na-MMT concentration rises (Figure 4c,d, Table 2).

**Figure 5:**
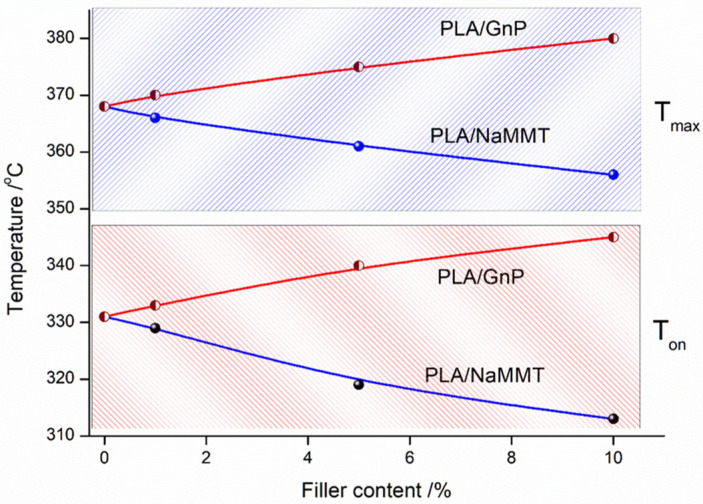
Concentration-dependent variation in experimental onset temperature (T_on_) and peak temperature (T_max_) for pristine PLA and PLA-based composites containing GnP or Na-MMT fillers.

The trends become particularly evident in Figure 5, which plots how the experimental T_on_ and T_max_ values vary with the concentration of fillers (GnP and Na-MMT) added to the PLA polymer matrix.

It is evident that the Na-MMT modifier plays a crucial role in promoting the PLA depolymerization through an unzipping mechanism. This process appears to engage both hydroxyl and carbonyl functional groups in the polymer structure. Additionally, an alternative reaction pathway involving interchain transesterification may be activated. Consequently, the presence of Na-MMT in PLA compositions leads to a noticeable decrease in thermal stability. In contrast, the thermal stabilizing effect of GnP in PLA composites is demonstrated by increased onset (*T_on_*) and maximum (*T_max_*) degradation temperatures. Two primary mechanisms contribute to this improvement: (1) robust interfacial interactions between GnP and PLA that limit the mobility of polymer chains in the molten degradation phase, and (2) the formation of a physical barrier by GnP particles that hinders both heat propagation and mass transfer, ultimately reducing material loss during thermal decomposition.

### 3.4. Pyrolysis–Gas Chromatography–Mass Spectrometry (Py-GC/MS) of PLA, PLA/Na-MMT, and PLA/GnP Compositions

**Figure 6:**
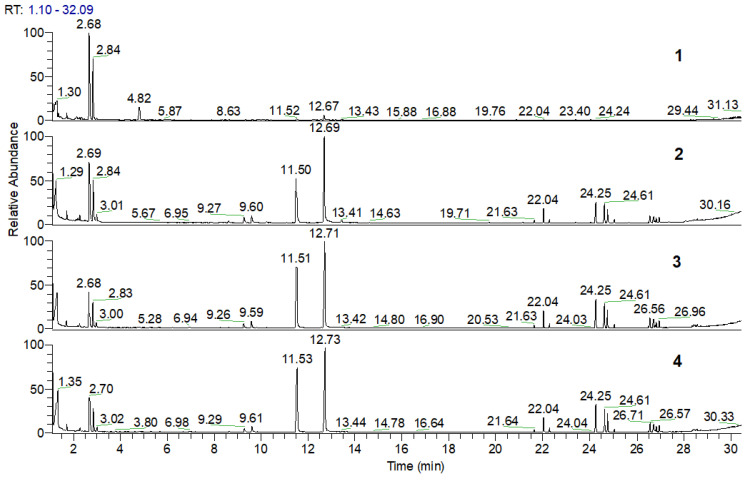
Chromatograms of pyrolysis products for pristine PLA (1), PLA/Na-MMT 1 wt.% (2), PLA/Na-MMT 5 wt.% (3), and PLA/Na-MMT 10 wt.% (4).

**Figure 7:**
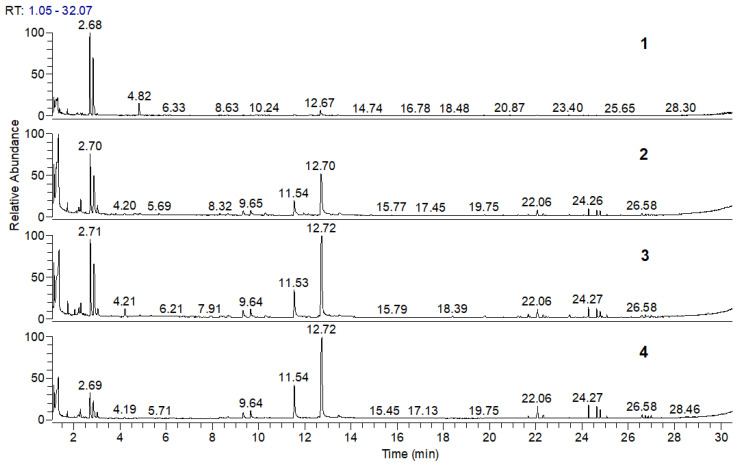
Chromatograms of pyrolysis products for PLA (1), PLA/GnP 1 wt.% (2), PLA/GnP 5 wt.% (3), and PLA/GnP 10 wt.% (4).

Pyrolysis of PLA, PLA/Na-MMT, and PLA/GnP composites was performed at 400 °C in Ar, which corresponds to the final stage of thermal degradation as evidenced by the TG curves (Figure 4). The resulting pyrolysis products were analyzed using gas chromatography coupled with mass spectrometric detection (GC/MS). Figure 6 and Figure 7 presents chromatograms of the pyrolysis products for pure PLA, PLA/Na-MMT and PLA/GnP composites containing 1, 5, and 10 wt.% filler. The corresponding composition data are provided in Table 3 and Table 4. Analysis of the data presented in Table 3 and Table 4 reveals that lactides and dioxalanones represent the predominant pyrolysis products generated from both PLA and its composite formulations. This data corroborates findings from prior studies of Usachev et al. [27] where nanolayered clay and nanographite additives significantly modify the thermal degradation behavior of PLA. These results demonstrated that such layered materials substantially modified the balance of the main pyrolysis products (lactides and 1,3-dimethyldioxolan-4-ones).

**Figure 8:**
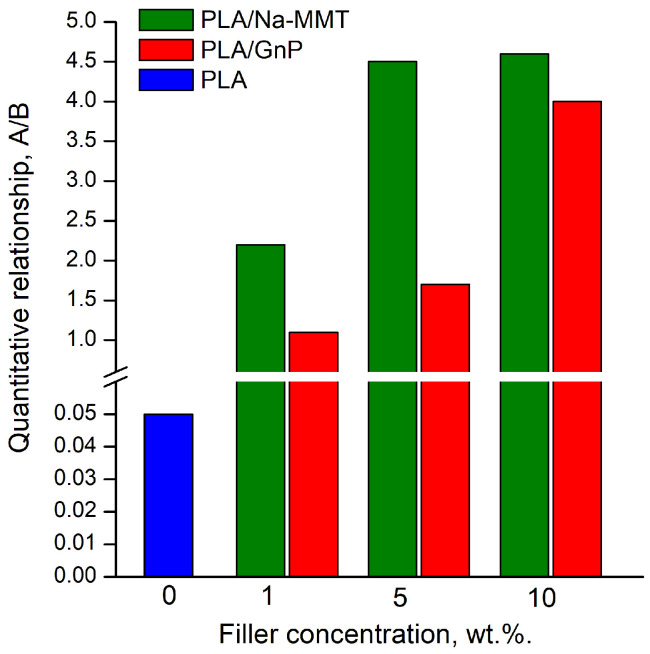
The quantitative relationship of lactides to 1,3-dimethyldioxolan-4-ones (A/B).

The content analysis of PLA composite pyrolysis products (Table 5 and Figure 8) revealed that increasing filler concentration (Na-MMT, GnP) in PLA composites alters the ratio of primary thermal degradation products. Specifically, in both PLA/Na-MMT and PLA/GnP systems, the proportion of five-member cyclic 1,3-dimethyldioxolan-4-ones decreases, while that of six-member cyclic lactides increases. Notably, the elevating lactide levels in thermal degradation products and the varying the (A)/(B) proportion is considerably stronger in PLA/Na-MMT than PLA/GnP composites. The layered aluminosilicate Na-MMT appears to exert catalytic influence on the PLA degradation process. When filler concentration increases to 10 wt.%, the quantitative (A)/(B) relationship becomes nearly equivalent in both composite types. This equalization is presumably due to the hindering of PLA macromolecules mobility induced by the fillers’ layered configuration [28].

According to the previous study [28], thermal degradation of PLA primarily results in the formation of two types of cyclic compounds: five-membered 1,3-dimethyldioxolan-4-ones and six-membered lactides.

**Figure 9:**
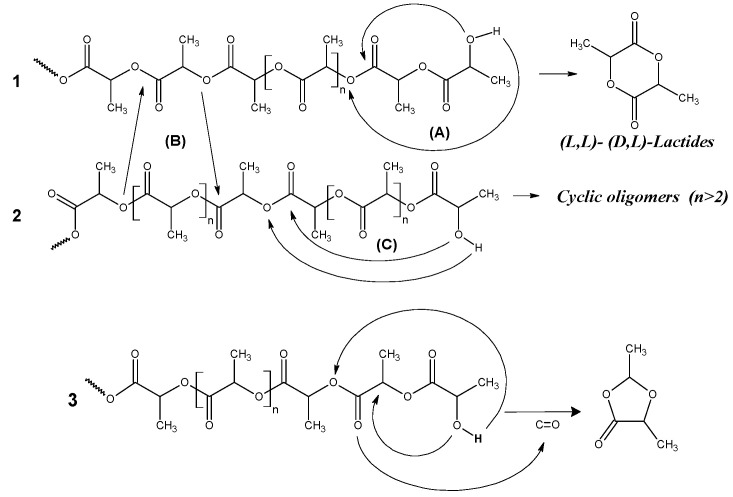
Formal schemes of the formation of lactides and *cis*-, *trans*-1,3-dimethyldioxalan-4-ones.

The proposed thermal degradation mechanism of PLA is schematically represented in Figure 9. The figure illustrates the following: (A) the unzipping depolymerization (designated 1) and (B) intermolecular reactions featuring end group (designated 2) routes to cyclic lactides; and a pathway leading to five-membered cyclic 1,3-dimethyldioxolan-4-ones (3).

### 3.5. Kinetic Analysis of Thermal Degradation for PLA, PLA/Na-MMT, and PLA/GnP Compositions

Nonlinear kinetic modeling of thermal degradation is a crucial aspect in understanding the stability and decomposition behavior of polymers such as polylactic acid (PLA) and its composites. This modeling approach involves analyzing how these materials break down under heat, which is essential for applications requiring thermal resistance and for optimizing processing conditions. The study typically compares experimental thermogravimetric analysis (TGA) data with theoretical model predictions to validate the kinetic parameters and degradation mechanisms. In the context of PLA, PLA/Na-MMT, and PLA/GnP, the analysis provides insights into how different fillers influence thermal stability.

MMT (montmorillonite) and graphene nanoplates (GnP) are commonly used to enhance the properties of PLA, including its thermal resistance. The comparison between experimental data (represented by dots) and model results (depicted by firm lines) at various heating rates allows researchers to evaluate the accuracy of the kinetic models and understand the degradation pathways. The kinetic models often involve complex mathematical equations that describe the rate of thermal degradation as a function of temperature and material composition. These models help in predicting the behavior of materials under different thermal conditions, which is vital for designing materials with specific performance criteria. The analysis also aids in identifying the activation energy and reaction mechanisms involved in the degradation process. Understanding the thermal degradation behavior through nonlinear kinetic modeling is essential for developing more durable and thermally stable polymer composites. It also contributes to the advancement of sustainable materials by optimizing their processing and end-use performance. Overall, this approach provides a comprehensive understanding of the thermal stability of PLA-based materials and their composites, guiding future research and industrial applications.

Research into the kinetics of material degradation dates back many years and has led to the development of numerous data analysis approaches. In practice, thermogravimetric analysis (TGA) is commonly used to collect experimental data for kinetic modeling—this technique was also adopted in the present study.

It is generally accepted that the degradation of materials follows the base Equation (1) [42]:

*dc*/*dt* = −*F*(*t*,*T*·*c_o_*·*c_f_*), (1)

where *t*—time; *T*—temperature; *c_o_*—initial concentration of the reactant; and *c_f_*—concentration of the final product.

Mathematically, the right-hand term of the equation *F*(*t*,*T*,*c_o_*,*c_f_*) decomposes into two independent functions: a temperature-dependent term *k*(*T*) and a concentration-dependent term *f*(*c_o_*,*c_f_*):

*F*(*t*,*T*,*c_o_*,*c_f_*) = *k*[*T*(*t*)·*f*(*c_o_*,*c_f_*)], (2)

The Arrhenius Equation (4) is assumed to be valid for the following:

*k*(*T*) = *A*·*exp*(−*E*/*RT*), (3)

where *A* is the pre-exponential factor and *E* is the activation energy.

Therefore,

*dc*/*dt* = −*A*·*exp*(−*E*/*RT*)·*f*(*c_o_*,*c_f_*), (4)

All possible reactions fall into two main categories, namely classical homogeneous reactions and typical solid-state reactions, which are summarized in Table 6. The analytical results of thermal kinetic analysis should demonstrate a good fit to experimental data obtained under various temperature profiles using a unified kinetic model.

Kinetic analysis of thermal degradation of PLA, PLA/Na-MMT 10 wt.%, and PLA/GnP 10 wt.% at heating rates of 5, 10, and 20 K/min was accomplished using the interactive model-based nonlinear regression fitting approach in accordance with a formalism proposed earlier [36]. Regression values were calculated using the fifth-degree Runge–Kutta method for approximate solutions for ordinary differential equations (ODEs) with the built-in Prince–Dormand formula for automatic optimization of the number of significant digits using the NETZSCH Thermokinetic 3 software.

In order to assess the activation energy for development of a reasonable model for kinetic analysis of PLA, PLA/Na-MMT, and PLA/GnP thermal degradation processes, a few evaluations by model-free Ozawa analysis have been performed as the starting point [43].

Since in these studies of the composite thermal degradation a dependence of the residual mass on the heating rate was observed (Figure 1, Figure 2 and Figure 3), a model of branched type reactions was proposed.

**Figure 10:**
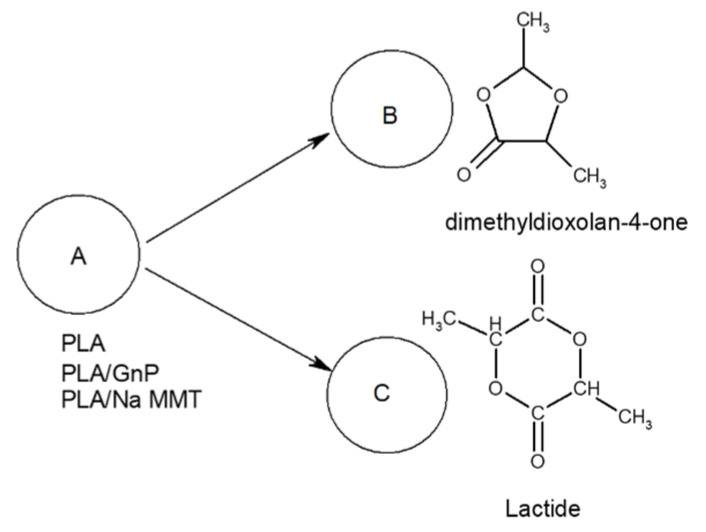
Model kinetic scheme of PLA, PLA/Na-MMT, and PLA/GnP thermal degradation.

Furthermore, nonlinear model fitting for PLA, PLA/Na-MMT, and PLA/GnP TGA-curves has led to the following two-stage model scheme of completive reactions (Figure 10).

Thermal degradation of PLA and PLA/Na-MMT was also assumed to follow first-order kinetics across all competitive stages, as secondary processes are unlikely at ~350 °C (peak of maximum degradation temperature; Table 2).

With the best fidelity, the nonlinear model two-stage model scheme of reactions, wherein general first-order (F_1_) reactions were used for PLA thermal degradation and first-order autocatalysis reactions (C_1_), was used for PLA/Na-MMT and two completive Avrami–Erofeev (A_n_)-type reactions for the PLA/GnP thermal degradation.

**Figure 11:**
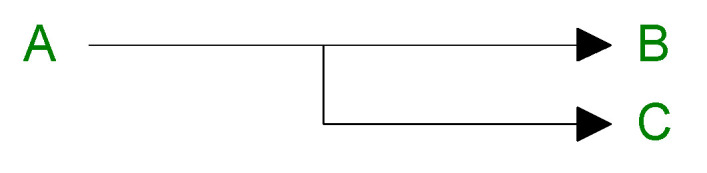
A formal scheme of two reactions competing process.

This model can be expressed through a system of differential equations (Figure 11).

$$\frac{da}{dt}=-A_{1}\times \exp(-\frac{E_{1}}{RT})\times f_{1}(a,b)-A_{2}\times \exp(-\frac{E_{2}}{RT})\times f_{2}(a,b)$$

$$\frac{db}{dt}=A_{1}\times \exp(-\frac{E_{1}}{RT})\times f_{1}(a,b)$$

$$\frac{dc}{dt}=A_{2}\times \exp(-\frac{E_{2}}{RT})\times f_{2}(a,c)$$

a=1−b−c

where *f*_1_ (*a*,*b*) and *f*_2_ (*a*,*b*) are reaction types corresponding to *F*_1_ (first-order reaction); *C*_1_ is the first-order reaction with autocatalysis); *A_n_* (n-dimensional nucleation, AVRAMI–EROFEEV) is used for reaction model equations.

**Figure 12:**
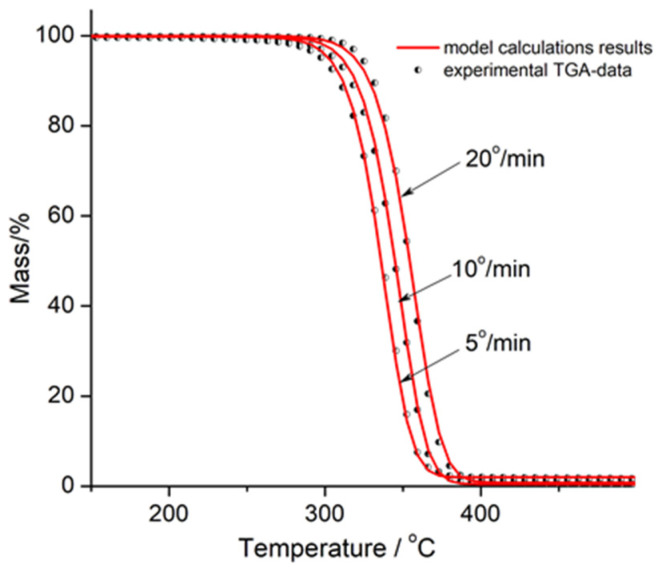
Nonlinear fitting results from multiple models for PLA. Experimental TGA data (dots) are compared with model predictions (solid lines) at heating rates of 5, 10, and 20 K/min.

**Figure 13:**
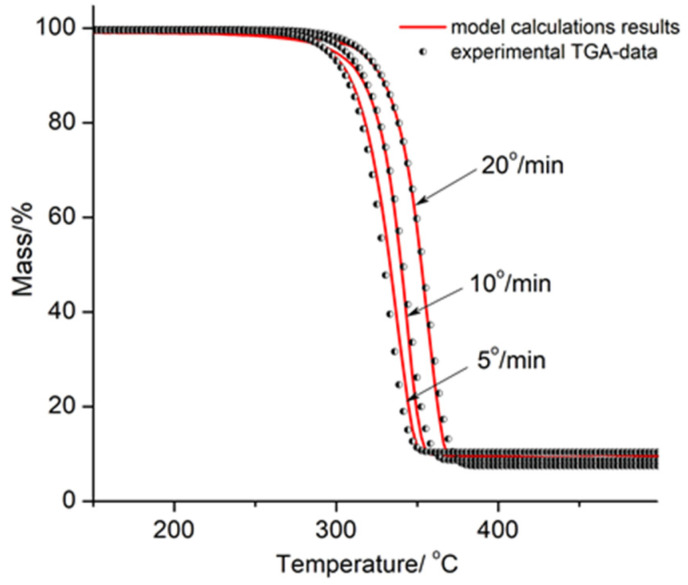
Nonlinear fitting results from multiple models for PLA/Na-MMT 10 wt.%. Experimental TGA data (dots) are compared with model predictions (solid lines) at heating rates of 5, 10, and 20 K/min.

**Figure 14:**
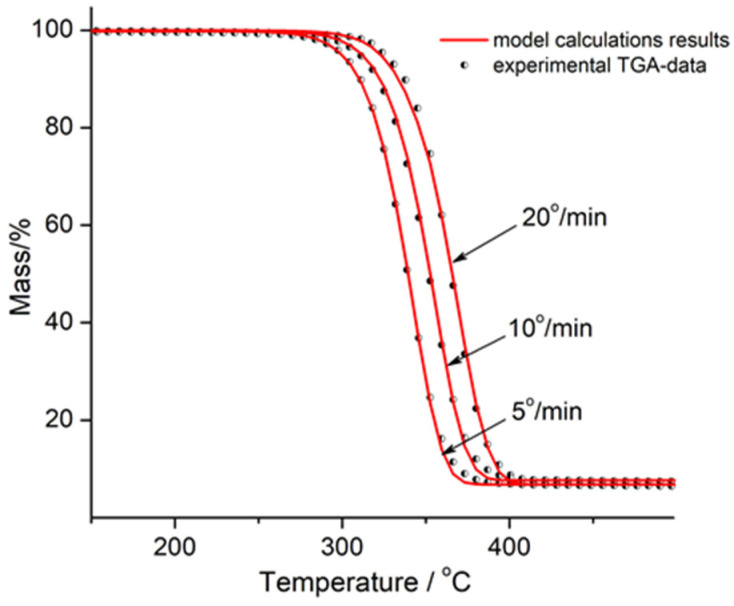
Nonlinear fitting results from multiple models for PLA/GnP 10% wt. Experimental TGA data (dots) are compared with model predictions (solid lines) at heating rates of 5, 10, and 20 K/min.

Figure 12, Figure 13 and Figure 14 show the graphical outcomes of applying nonlinear fitting model to PLA, PLA/Na-MMT, and PLA/GnP thermal degradation. All the figures display experimental thermogravimetric analysis (TGA) results as discrete data points (dots), alongside the corresponding model-predicted curves (solid lines) for three different heating rates: 5 K/min, 10 K/min, and 20 K/min.

Table 7 contains the principal kinetic parameters for the composite’s thermal degradation process, namely activation energies and pre-exponential factors.

Model Step 1 refers to the reaction pathway yielding 1,3-dimethyldioxalan-4-ones, whereas Model Step 2 denotes the reaction leading to lactide formation (see Figure 10).

Data analysis revealed that activation energies and pre-exponential factors at Model Step 1 are lower than those in the Model Step 2 stage of yielding lactides. For pristine PLA, activation energy rises notably between stages *E*_1_ = 176.8 kJ/mol (Model Step 1) and *E*_2_ = 229.3 kJ/mol (Model Step 2).

Table 7 shows similar trends for PLA/Na-MMT and PLA/GnP: activation energies increase from Model Step 1 to Model Step 2, though the rise is less pronounced than for pure PLA. This finding aligns with Py-GC/MS data (Figure 12), which demonstrate a shift in the lactide-to-1,3-dimethyldioxolan-4-one ratio (A:B) toward higher lactide concentrations in the total volatile degradation products compared to neat PLA.

In the present work, isothermal degradation simulations (predictions) at a constant temperature of 400 °C were conducted for PLA, PLA/Na-MMT 10% wt., and PLA/GnP 10%wt. These predictions were obtained by solving the forward kinetics problem using the input kinetic parameters obtained from the model kinetic analysis presented in Table 7. These calculations were conducted to establish a correlation between the quantitative Py-GC/MS results and the calculated values obtained from the kinetic accumulation curves of the five-membered cyclic 1,3-dimethyldioxolan-4-ones and six-membered cyclic lactides formed during sample pyrolysis at 400 °C.

**Figure 15:**
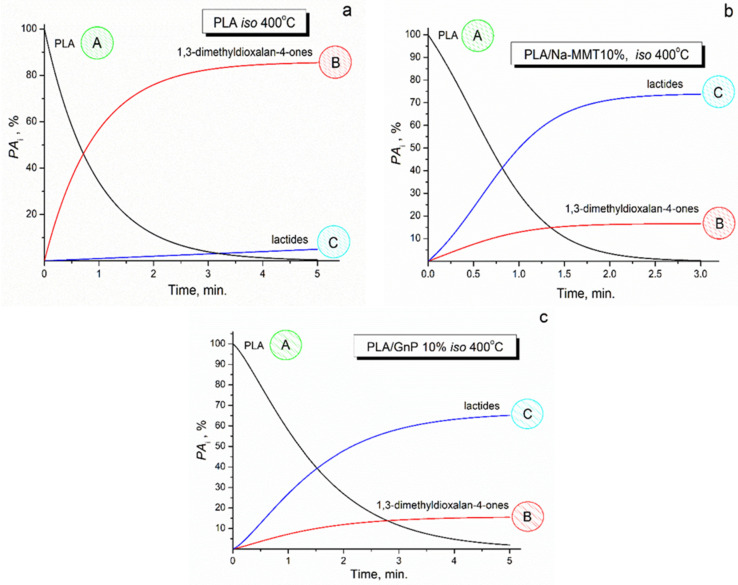
Calculated kinetic curves of consumption and accumulation under isothermal pyrolysis conditions at 400 °C for PLA (**a**), PLA/Na-MMT 10 wt.% (**b**), and PLA/GnP 10 wt.% (**c**), where PLA—A, 1,3-dimethyldioxolan-4-ones—B, and lactides—C.

The graphical representation in Figure 15 provides quantitative data on the kinetic behavior of thermal degradation processes at 400 °C, with separate curves depicting the consumption kinetics of PLA (kinetic curve A), the accumulation dynamics of 1,3-dimethyldioxolan-4-ones (kinetic curve B), and the formation kinetics of lactides (kinetic curve C).

Numerical validation of the computed isothermal kinetic parameters was performed by cross-referencing them with the experimental Py-GC/MS dataset (the concentrations of five-membered cyclic 1,3-dimethyldioxolan-4-ones and six-membered cyclic lactides formed during sample pyrolysis at 400 °C), as presented in Table 3 and Figure 9.

Fair agreement between model-predicted kinetics results and Py-GC/MS experimental data strongly supports the validity and practical utility of the proposed kinetic modeling approach.

## 4. Conclusions

The study uncovered distinct mechanisms by which two different additives, such as Na-MMT and GnP, affect the thermal degradation of PLA. When Na-MMT was introduced into the PLA matrix, it demonstrated a catalytic effect, significantly accelerating the material’s thermal destabilization. This acceleration was evident through faster degradation kinetics and a noticeable shift in the composition of the resulting pyrolysis products. In contrast, the addition of graphene nanoplates (GnP) produced a barrier effect. The GnP particles created diffusion limitations that impeded the release of volatile degradation products during pyrolysis. This increased the thermal stability of the PLA/GnP composite and led to quantitative changes in the distribution of the main pyrolysis by-products. To gain deeper insight into these degradation mechanisms, the model kinetic analysis of thermal degradation for both composite systems (PLA/GnP and PLA/Na-MMT) was conducted. The reliability of the kinetic modeling approach was substantiated by the strong correlation between the theoretical predictions and the experimental data. The latter was acquired using Py–GC/MS analysis.

An in-depth study employing TGA, DSC, Py-GC/MS, AFM, thermokinetic modeling, and prediction methods were conducted on PLA, PLA/Na-MMT, and PLA/GnP composites. The results provide valuable insights into the impact of graphene and clay fillers on the structural properties, thermal stability, and thermal degradation mechanisms of PLA composites, leading to several important findings:

1. Morphological analysis using AFM showed successful incorporation of fillers into the PLA matrix. Na-MMT demonstrated intercalation and exfoliation processes, while GnP formed interconnected layers with preferential orientation.
2. Thermal behavior investigation via DSC revealed the following:

Both fillers Na-MMT and GnP acted as nucleating agents, increasing PLA crystallinity;

Na-MMT showed more significant nucleating effect on crystallinity of PLA than GnP (up to 39.7%).

3. Thermal stability assessment using TGA demonstrated the following:

Na-MMT decreased thermal stability with increasing concentration by catalytical effect of Na-MMT on PLA depolymerization through an unzipping mechanism; in contrast to Na-MMT, GnP improved thermal stability of PLA composition (growth of T_on_ and T_max_) due to the formation of a physical barrier by graphene GnP particles that hinders mass transfer, ultimately reducing material loss by the volatilizing of degradation products during thermal decomposition.

4. Degradation mechanism study by Py-GC/MS showed the following:

Formation of lactides and dioxalanones as main degradation products; change in product ratio depending on filler type and concentration; different effects of Na-MMT and GnP on degradation pathways of PLA.

5. Kinetic modeling has successfully described the thermal degradation behavior of PLA-based materials through the formal scheme of two-stage competing reactions where
  the first stage produces 1,3-dimethyldioxalan-4-ones; the second stage leads to lactide formation; PLA follows first-order kinetics; PLA/Na-MMT exhibits first-order autocatalytic reactions; PLA/GnP shows Avrami–Erofeev-type kinetics;
  GnP and Na-MMT fillers modify the degradation mechanism;
  both fillers influence the ratio of degradation products.

6. The developed kinetic model provides the following:

Accurate prediction of thermal degradation behavior; quantitative description of reaction mechanisms; insight into the influence of fillers on degradation processes.

Validation results confirm the reliability of the model, providing good agreement between theoretical predictions and experimental data of Py-GC/MS, as evidenced by high values of correlation coefficients (up to 0.999889) and consistent Durbin–Watson statistics.

The results obtained offer a solid foundation for designing thermally stable PLA-based materials with tailored degradation properties for specific applications.

## Figures and Tables

**Table 1:** Calorimetric data derived from primary and secondary heating of PLA, PLA/Na-MMT, and PLA/GnP composites.

Sample	Heating	*T_g_* (°C)	*T_cc_* (°C)	*T_m_* (°C)	Δ*H_cc_* (J/g)	Δ*H_m_* (J/g)	χ (%)
PLA	*first*	56.6	114	163.2	2.0	−30.8	30.8
	*second*	61.3	n/a	158	n/a	−0.6	0.7
PLA/GnP 1 wt.%	*first*	54.5	113.1	162.4	1.8	−31.8	32.2
	*second*	61.3	128.6	159.0	7.1	−10.9	4.0
PLA/GnP 5 wt.%	*first*	64.6	110.5	162.8	5.8	−31.6	27.7
	*second*	61.1	127.1	158.5	16.5	−23.4	7.8
PLA/GnP 10 wt.%	*first*	53.9	113.2	162.2	n/a	−32.1	34.2
	*second*	62.0	121.3	157/162 ^1^	25.5	−32.2	3.9
PLA/Na-MMT 1 wt.%	*first*	51.7	n/a	163.5	n/a	−36.8	39.7
	*second*	61.2	n/a	158.3	n/a	−0.8	0.9
PLA/Na-MMT 5 wt.%	*first*	51.0	n/a	159.1	n/a	−35.5	39.0
	*second*	61.1	133.2	159.1	0.4	−2.0	1.9
PLA/Na-MMT 10 wt.%	*first*	66.4	113.9	160.8	2.7	−27.2	29.0
	*second*	60.8	132.8	158.1	0.9	−3.4	3.0

^1^ refer to the α′-limit disordered (hexagonal) crystalline forms of PLA.

**Table 2:** TGA results of PLA, PLA/Na-MMT, and PLA/GnP compositions.

Sample	T_on_ (°C)	T_max_ (°C)
PLA	331	368
PLA/GnP 1 wt.%	333	372
PLA/GnP 5 wt.%	340	375
PLA/GnP 10 wt.%	345	380
PLA/Na-MMT 1 wt.%	329	366
PLA/Na-MMT 5 wt.%	319	361
PLA/Na-MMT 10 wt.%	313	356

**Table 3:** The content of pyrolysis products (PAi) of PLA and PLA/Na-MMT with a filler content of 1, 5, and 10 wt.%, respectively.

Retention Time (min)	Pyrolysis Products	*PA_i_* (wt.%)			
		0	1	5	10
1.32	Acrylic acid	9.10	8.90	8.00	9.83
2.15	Vinylacetic acid	1.48	0.80	0.36	0.23
2.65	*cis*-1,3-dimethyldioxolan-4-one	49.03	15.29	9.18	8.99
2.8	*trans*-1,3-dimethyldioxolan-4-one	34.60	10.65	6.45	6.09
11.48	*meso*-lactide	0.85	11.29	15.54	16.92
12.69	*D*,*L*-lactide	2.85	21.87	22.04	22.06
21.6 ÷ 22.2	Trimer (*n* = 3)	0.45	5.23	6.32	5.54
24.2 ÷ 24.7	Tetrame (*n* = 4)	0.54	13.56	18.64	18.30
26.5 ÷ 26.9	Pentamer (*n* = 5)	0.00	5.88	8.34	7.56
	Unidentified compounds	1.11	6.52	5.12	4.47

**Table 4:** The content of pyrolysis products (PAi) of PLA and PLA/GnP with a filler content of 1, 5, and 10 wt.%, respectively.

Retention Time (min)	Pyrolysis Products	*PA_i_* (wt.%)			
		0	1	5	10
1.36	Acrylic acid	9.10	25.91	16.39	13.33
2.19	Vinylacetic acid	1.48	2.13	2.25	1.06
2.7	*cis*-1,3-dimethyldioxolan-4-one	49.03	21.70	20.86	9.14
2.85	*trans*-1,3-dimethyldioxolan-4-one	34.60	14.01	14.10	6.43
11.53	*meso*-lactide	0.85	5.26	7.21	11.78
12.71	*D*,*L*-lactide	2.85	14.56	21.72	28.40
21.6 ÷ 22.2	Trimer (*n* = 3)	0.45	3.06	3.78	6.17
24.2 ÷ 24.7	Tetrame (*n* = 4)	0.54	6.23	6.34	12.36
26.5 ÷ 26.9	Pentamer (*n* = 5)	0.00	2.20	1.91	4.33
	Unidentified compounds	1.11	4.95	5.43	6.98

**Table 5:** The quantitative relationship of lactides (A) to 1,3-dimethyldioxolan-4-ones (B).

Composition	The Quantitative Relationship (A):(B)
PLA	1.0:18.0
PLA/Na-MMT 1 wt.%	2.2:1.0
PLA/Na-MMT 5 wt.%	4.5:1.0
PLA/Na-MMT 10 wt.%	4.6:1.0
PLA/GnP 1 wt.%	1.1:1.0
PLA/GnP 5 wt.%	1.7:1.0
PLA/GnP 10 wt.%	4.0:1.0

**Table 6:** Reaction types and corresponding reaction equations, *dc*/*dt*= −*A*·*exp*(−*E*/*RT*)·*f*(*c_o_*,*c_f_*).

Name	*f*(*c_o_*,*c_f_*)	Reaction Type
F_1_	*c*	first-order reaction
F_2_	*c^2^*	second-order reaction
F_n_	*c^n^*	*n*th-order reaction
R_2_	2·*c*^1/2^	two-dimensional phase boundary reaction
R_3_	3·*c*^2/3^	three-dimensional phase boundary reaction
D_1_	0.5/(1 − *c*)	one-dimensional diffusion
D_2_	−1/*ln*(*c*)	two-dimensional diffusion
D_3_	1.5·*e*^1/3^(*c*^−1/3^ − 1)	three-dimensional diffusion (Jander’s type)
D_4_	1.5/(*c*^−1/3^ − 1)	three-dimensional diffusion (Ginstling–Brounstein type)
B_1_	*c_o_·c_f_*	simple Prout–Tompkin’s equation
B_na_	*c_o_^n^·c_f_^a^*	expanded Prout–Tompkin’s equation (n_a_)
C_1-X_	*c·*(1 + *K_cat_·X*)	first-order reaction with autocatalysis through the reactants, *X*. *X* = *c_f_*
C_n-X_	*c^n^·*(1 + *K_cat_·X*)	*n*th-order reaction with autocatalysis through the reactants, *X*
A_2_	2·*c·*(−*ln*(*c*))^1/2^	two-dimensional nucleation
A_3_	3·*c*·(−*ln*(*c*))^2/3^	three-dimensional nucleation
A_n_	*N·c·*(−*ln*(*c*))^(*n*−1)/*n*^	*n*-dimensional nucleation/nucleus growth according to Avrami/Erofeev

**Table 7:** Results of the multiple-curve kinetic analysis for thermal degradation of PLA, PLA/Na-MMT, and PLA/GnP in accordance with the reaction model (2).

Composition	Model Reaction	Parameter	Value	Statistics
PLA	Model Step 1: first-order reaction	*lgA*_1_, s^−1^ *E*_1_, kJ/mol	10.7 176.8	*Correlation coefficient: 0.999388* *Durbin*–*Watson* *Value: 0.357*
	Model Step 2: first-order reaction	*lgA*_2_, s^−1^ *E*_2_, kJ/mol	16.9 229.3	
PLA/Na-MMT 10 wt.%	Model Step 1: first-order reaction with autocatalysis	*lgA*_1_, s^−1^ *E*_1_, kJ/mol *lgK_cat_*	11.1 170.1 0.5	*Correlation coefficient: 0.999402* *Durbin*–*Watson* *Value: 0.081*
	Model Step 2: first-order reaction with autocatalysis	*lgA*_2_, s^−1^ *E*_2_, kJ/mol *lgK_cat_*	11.7 198.0 0.8	
PLA/GnP 10 wt.%	Model Step 1: n-dim. Avrami–Erofeev	*lgA*_1_, s^−1^ *E*_1_, kJ/mol *dimention*_1_	9.5 142.3 1.20	*Correlation coefficient: 0.999889* *Durbin*–*Watson* *Value: 0.272*
	Model Step 2: n-dim. Avrami–Erofeev	*lgA*_2_, s^−1^ *E*_2_, kJ/mol *dimention*_2_	12.5 178.6 1.13	

## Data Availability

The data presented in this study are available on request from the corresponding authors.
